# Modeling Atrial Fibrillation using Human Embryonic Stem Cell-Derived Atrial Tissue

**DOI:** 10.1038/s41598-017-05652-y

**Published:** 2017-07-13

**Authors:** Zachary Laksman, Marianne Wauchop, Eric Lin, Stephanie Protze, Jeehoon Lee, Wallace Yang, Farzad Izaddoustdar, Sanam Shafaattalab, Lior Gepstein, Glen F. Tibbits, Gordon Keller, Peter H. Backx

**Affiliations:** 1 University of British Columbia, Vancouver, Canada; 2 University of Toronto, Toronto, Canada; 3 Simon Fraser University, Burnaby, Canada; 4 McEwen Center of Regenerative Medicine, Toronto, Canada; 5 McEwen Center for Regenerative Medicine, Toronto, Canada; 6 University of Toronto, Toronto, Canada; 7 University of Toronto, Toronto, Canada; 8 Simon Fraser University, Burnaby, Canada; 9 Cardiac Electrophysiology and Regenerative Medicine, Technion, Haifa Israel; 10 Simon Fraser University, Burnaby, Canada; 11 McEwen Center for Regenerative Medicine, Toronto, Canada; 12 York University, Toronto, Canada

## Abstract

Since current experimental models of Atrial Fibrillation (AF) have significant limitations, we used human embryonic stem cells (hESCs) to generate an atrial-specific tissue model of AF for pharmacologic testing. We generated atrial-like cardiomyocytes (CMs) from hESCs which preferentially expressed atrial-specific genes, and had shorter action potential (AP) durations compared to ventricular-like CMs. We then generated confluent atrial-like CM sheets and interrogated them using optical mapping techniques. Atrial-like CM sheets (~1 cm in diameter) showed uniform AP propagation, and rapid re-entrant rotor patterns, as seen in AF could be induced. Anti-arrhythmic drugs were tested on single atrial-like CMs and cell sheets. Flecainide profoundly slowed upstroke velocity without affecting AP duration, leading to reduced conduction velocities (CVs), curvatures and cycle lengths of rotors, consistent with increased rotor organization and expansion. By contrast, consistent with block of rapid delayed rectifier K+ currents (Ikr) and AP prolongation in isolated atrial-like CMs, dofetilide prolonged APs and reduced cycle lengths of rotors in cell sheets without affecting CV. In conclusion, using our hESC-derived atrial CM preparations, we demonstrate that flecainide and dofetilide modulate reentrant arrhythmogenic rotor activation patterns in a manner that helps explain their efficacy in treating and preventing AF.

## Introduction

Atrial fibrillation (AF), already the most common clinical arrhythmia, is expected to double in prevalence over the next 4 decades^1^. AF is associated with a doubling in all-cause mortality, a five-fold increase in stroke, an accelerated development of heart failure, and a substantially poorer quality of life^2, 3^. Ablation techniques have improved outcomes over the last 2 decades, but are associated with a risk of complications, and are only successful in a limited number of patients with permanent AF. Pharmacologic therapy remains as first-line treatment, and an important adjuvant to ablation therapy, however is limited by a lack of efficacy, ventricular pro-arrhythmia, and serious side effects^4, 5^. One of the major barriers to an improved mechanistic understanding of AF, and thus in the pipeline of drug development, has been a lack of appropriate models. The challenges of interspecies differences in receptor subtypes, distribution and signalling are compounded by significant differences in the ion channels and gap junctions that work in concert to dictate the pathophysiology of human AF^6–8^.

Human pluripotent stem cell (hPSC) derived cardiomyocytes (CM) represent a promising and powerful tool for anti-arrhythmic drug (AAD) screening, and have been exploited to model a growing number of arrhythmogenic cardiac electrical disorders at a single cell level^9^. Investigations have predominantly focused on diseases of the ventricles, in some part because current differentiation protocols produce predominantly ventricular-like CMs, with a small minority of atrial-like and nodal-like cells^10^. A major step forward has been the refinement and validation of differentiation protocols that can enrich the atrial-like population to screen for the effects of drugs on human atrial electrophysiology^11^. AF however cannot be modeled using single cells. Thus the major objective of our studies is to develop and validate a novel platform for AAD screening for the treatment of AF using hPSCs in multicellular constructs that can re-capitulate the macroscopic footprints of the human disease.

Through directed differentiation of human embryonic stem cells (hESCs), we generated CMs with largely (>85%) atrial-like properties, indicated by mRNA expression patterns and single CM action potential (AP) profiles^12, 13^. From the atrial-like CMs, we created multicellular sheets that beat spontaneously in response to depolarization from a single “pacemaker” site. We induced stable re-entrant spiral wave geometries (called rotors), which act as arrhythmogenic self-organized drivers in AF^14–16^, using rapid external pacing, and employed optical mapping techniques to study the electrical effects of two commonly used AADs (i.e. flecainide and dofetilide) for AF treatment. The actions of the AADs on selected ionic currents were also interrogated in voltage-clamp studies of isolated atrial-like CMs. Consistent with their ionic mechanisms, both flecainide and dofetilide altered rotor dynamics in a manner that helps explain their clinical actions. The significance and limitations of our findings are discussed in the context of AF and its treatment.

## Methods

### Directed differentiation of atrial-like CMs

Culture and differentiation of hESCs to atrial- and ventricular-like CMs employed protocols that have previously been reviewed in detail, and are available in the Supplementary Material^17, 18^. In brief, HES3 NKX2–5^egfp/w^ cells were differentiated into CMs using an embryoid body (EB) protocol. We used Activin A and BMP4 signaling for mesoderm induction followed by Wnt inhibition for cardiac specification (control protocol) (Supplemental Fig. 1). To drive CMs towards an atrial phenotype, we added retinoic acid (RA) at the cardiac mesoderm stage (atrial protocol)^11, 19^. The timing and concentrations of cytokines utilized were optimized using previously described flow cytometric analyses^12, 20–22^ (Supplemental Fig. 3)^13^. Molecular analyses of atrial and ventricular-like CMs were carried out using a qRT-PCR marker set. Details regarding flow cytometry, cell sorting, qRT-PCR, and immunostaining and microscopy can be found in the Supplementary Material.

### Single Cell Electrophysiology

EBs resulting from control and atrial differentiations were dissociated at day 20 using type B collagenase and TrypLE. Cells were sorted based on DAPI^−^/SIRPA^+^/NKX2–5/GFP^+^/CD90^−^ surface markers as previously described (and discussed in Supplement), generating a pure population of CMs. Electrophysiological measurements were performed 7–10 days after dissociation and plating. Spontaneous and stimulated action potentials (APs) were recorded using the patch clamp technique in the whole cell configuration^23^. Data was analyzed using Clamp fit (Molecular Devices, Sunnyvale, CA, U.S.A). For further details, see Supplementary Material.

### Preparation of cell sheets

Cell sheets were formed from dissociated EBs on day 20 of the atrial differentiation protocol. Cell sheets consisted of 1.5 million cells and formed sheets that were 1 cm in diameter. Flow cytometry was performed routinely to ensure >85% CM production efficiency by analyzing the percentage of cells that were DAPI-/SIRPA+/NKX2-5:GFP+/CD90−. Cell sheets were incubated under normoxic conditions at 37 °C for 2 weeks prior to imaging. Further details are provided in the Data Supplement.

### Optical mapping

We used the voltage sensitive dye AminoNaphthylEthenylPyridinium (Di-4-ANEPPS)(Life Technologies) at 10 μM^24^. Blebbistatin 10 μM (Sigma-Aldrich) was employed to avoid motion artifact^25^. Images were captured using an electron multiplying charge coupled device (EMCCD) camera (Cascade 128+, Cascade Evolve, Photometric, Tucson, AZ, U.S.A). Arrhythmia induction was carried out using rapid pacing protocols (median cycle length (CL) = 200 ms, range 150–300 ms) or burst pacing (CL of 50 ms for 30 seconds). The AADs flecainide (Sigma-Aldrich) and dofetilide (Sigma-Aldrich) were added to the culture dish in sequentially increasing concentrations after arrhythmia induction (see Supplement). Signal processing of the optically mapped data was performed using a custom IDL software program or Scroll software. Further details are provided in the Data Supplement.

Statistical Analysis was performed using Prism 6 for MacOS X (GraphPad Software Inc, 2014). Univariate testing was performed using the Student’s t -test or the Mann–Whitney U test. Paired data were compared using the paired t -test.

## Results

**Figure 1:**
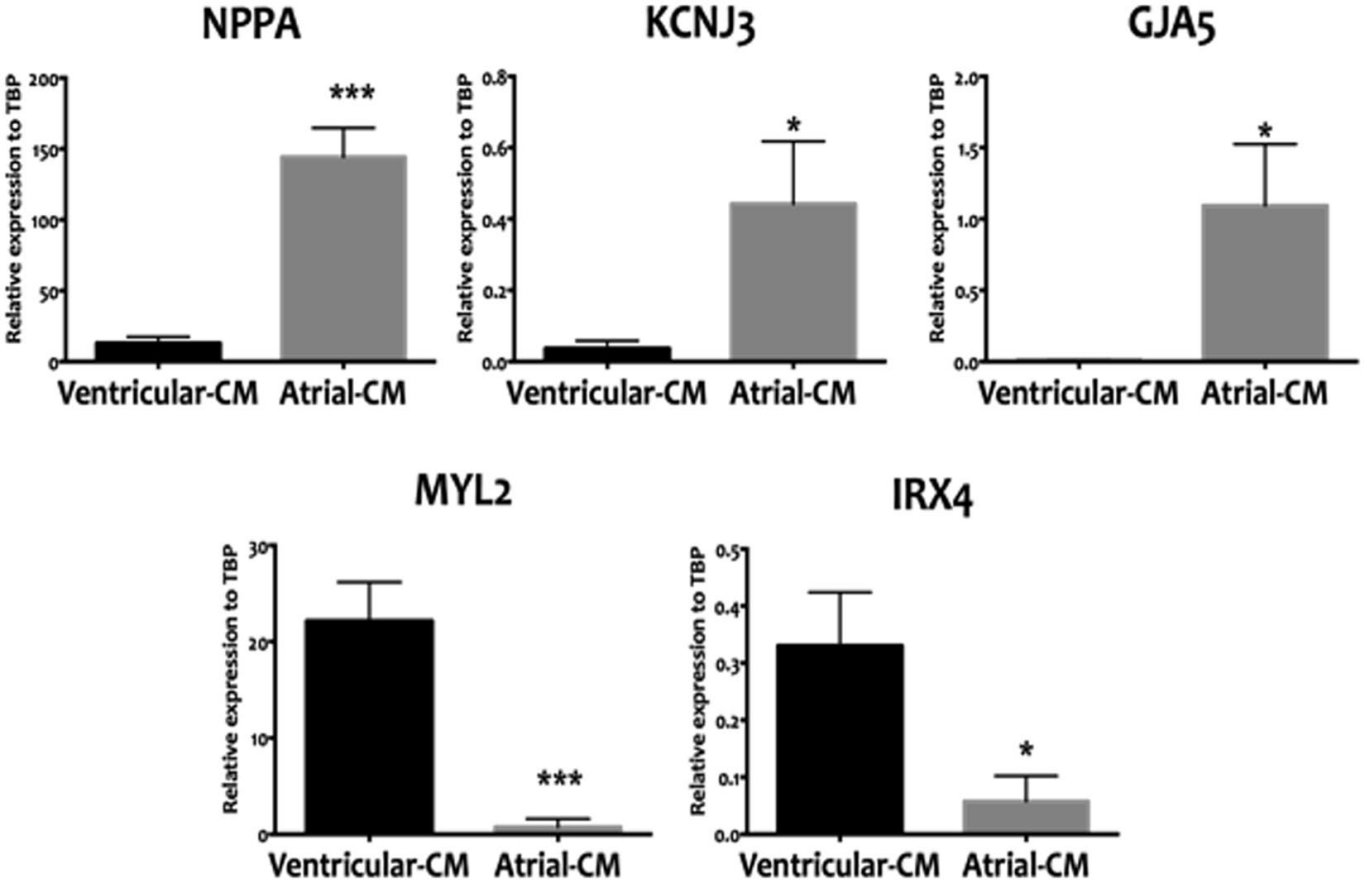
qRT-PCR-based expression analyses directly comparing the atrial differentiation protocol that employed RA (atrial-CM, grey bars) and ventricular differentiation protocol (ventricular-CM, black bars). Cell populations were analyzed at T20 of the differentiation protocol. The RA protocol was able to generate cells that are enriched in atrial specific markers (NPPA, KCNJ3, and GJA5) and lacked ventricular markers (MYL, IRX4). Values are shown relative the housekeeping gene TBP. Error bars represent standard deviation of the mean from the values of independent experiments (N ≥ 4); *P ≤ 0.05, **P ≤ 0.01, ***P ≤ 0.001.

Flow cytometric analyses were performed on hESCs before cardiac differentiation in order to ensure a high frequency of pluripotency markers and low frequency of differentiation (Supplemental Fig. 2) prior to cardiomyocyte induction. By employing previously validated marker sets^17, 18^, we then optimized cardiac differentiation and routinely generated >85% cardiac troponin T positive cells (Supplemental Fig. 3). As summarized in Fig. 1, further molecular characterization of the two differentiation protocols revealed that the RA-based atrial differentiation protocol, in comparison to the control protocol, generated cells that expressed higher (*P* ≤ 0.05) levels of the atrial-specific markers *NPPA* (natriuretic peptide type A), *KCNJ3* (the potassium inwardly-rectifying channel) and *GJA5* (connexin40). The control differentiation protocol similarly showed higher (*P* ≤ 0.05) levels of expression of ventricular specific markers such as the ventricular paralog of the regulatory light chain of myosin (*MYL2*), and the Iroquois-class homeodomain protein *IRX-4* (IRX4).

### Electrical characterization of isolated atrial-like CMs and cell sheets of atrial-like CMs

**Figure 2:**
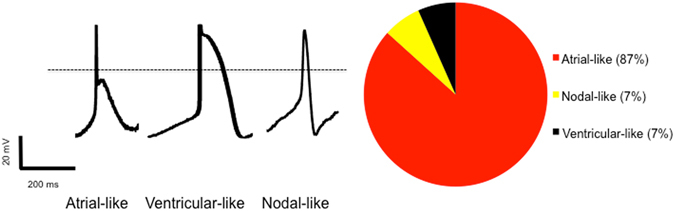
Single cell patch clamping studies of cardiomyocytes generated from the atrial differentiation protocol which employed retinoic acid (N = 15 cells). Representative action potentials displayed representing the three electrophysiologic phenotypes identified in the population. Right image displaying the prevalence of various AP morphologies in our cell cultures which demonstrates an overwhelming majority of atrial-like CMs.

The atrial differentiation protocol generated CMs in which ~87% showed atrial-like APs, with the remaining cells showing nodal-like (~7%) or ventricular-like (~7%) APs^26^. Our standard control differentiation protocol, that lacks RA, generated ~80% ventricular-like, ~13% atrial-like and 7% nodal-like APs (Fig. 2). CMs generated from the atrial differentiation protocol had AP durations at 30% (APD30) and 90% (APD90) repolarization of 37 ± 11 ms and 247 ± 37 ms, respectively. Moreover, the ratio of APD30 to APD90 in CMs from our atrial differentiation protocol (APD30/APD90 = 0.17 ± 0.04) are consistent with that seen in adult atrial CMs^27^ and differs (P < 0001) from the ratio seen in our standard differentiation protocol (APD30/APD90 = 0.57 ± 0.07) as summarized in Table 1. Despite showing AP properties typical of adult atrial CMs, the CMs generated with our atrial differentiation protocol displayed electrophysiologic properties indicative of immaturity, including a relatively positive maximum diastolic potential, slow maximum upstroke velocities, and small action potential amplitudes (Table 1)^28, 29^. To examine whether the slow upstroke velocities arose from the loss of functional Na^+^ current resulting from depolarized diastolic potentials, we performed AP-clamp studies. Specifically, cells were voltage-clamped with the holding potential being either the average diastolic membrane potentials observed in the atrial CMs (i.e. −56 mV, Table 1) or a holding potential of −80 mV, a value typical of adult human atrial CMs^28, 29^. Consistent with the strong dependence of I_Na_ inactivation on the diastolic potential, we found that I_Na_ was reduced by 40.43 ± 5.49% (n = 10) when the holding potential was −58 mV versus a holding potential of −80 mV.

**Table 1:** Single-cell patch-clamp studies comparing the control differentiation protocol to the atrial protocol which employed retinoic acid.

	N (cells)	dV/dt_max_ (V/s)	DMP (mV)	APA (mV)	APD30 (ms)	APD90 (ms)
Control Differentiation protocol	15	62 ± 9	−53 ± 2	102 ± 5	327 ± 98	520 ± 120
Atrial Differentiation protocol	15	63 ± 13	−56 ± 2	80 ± 5**	37 ± 11**	247 ± 37*

Most notable, and consistent with the adult phenotype, cardiomyocytes generated from the atrial differentiation protocol had dramatically shorter repolarization times. Cardiomyocytes generated from both protocols display features of immaturity as discussed in the text. dV/dt_max_ = Maximum action potential upstroke velocity, DMP = Diastolic membrane potential, APA = Action potential amplitude, APD30 = Action potential duration at 30% of repolarization, APD90 = Action potential duration at 90% of repolarization. *P < 0.05, **P < 0.01.

**Figure 3:**
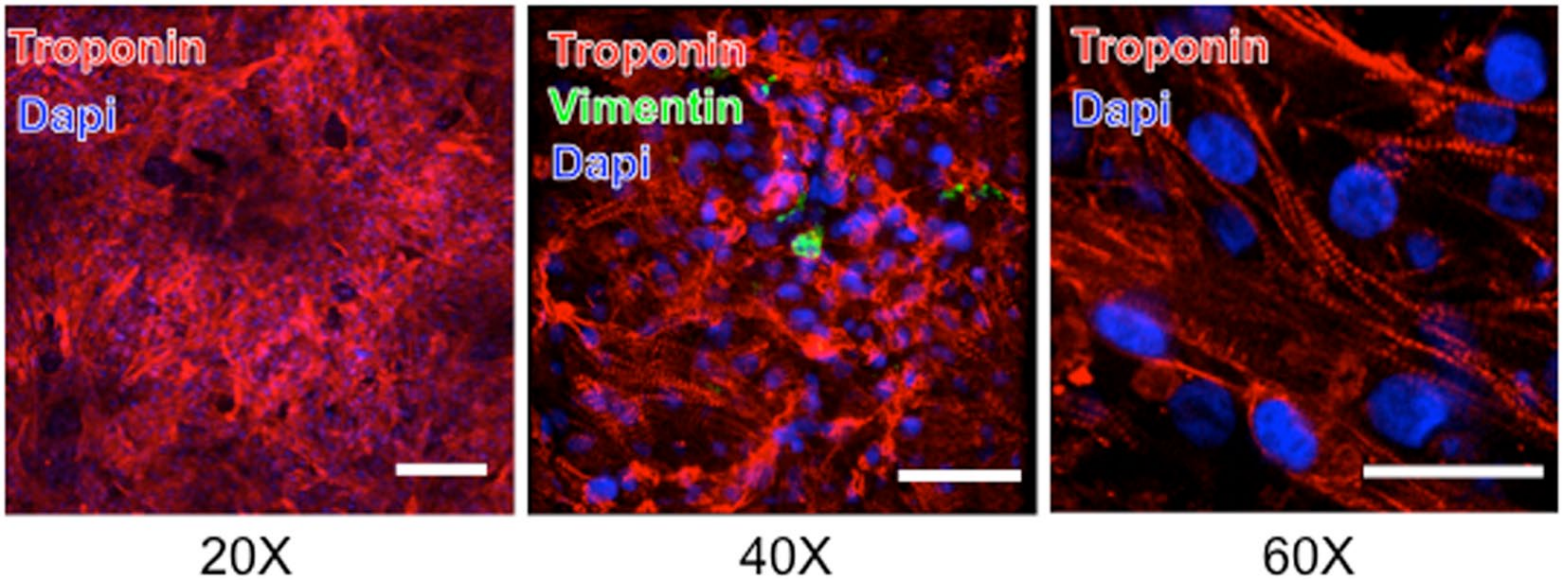
Fluorescent immunostaining for the presence of troponin and vimentin in cell sheets generated from the atrial differentiation protocol. DAPI staining shows cell nuclei. Troponin staining demonstrates disorganized ultrastructural arrangement of cardiomyocytes. Vimentin staining showing infrequent and isolated mesenchymal cells. Scale bar is 50 μm.

We next generated confluent cell sheets of atrial CMs that had a diameter of ~1 cm. These cell sheets beat spontaneously and synchronously within 24 hours of plating, and immunostaining revealed well developed cardiac myofilaments with <1% Vimentin + mesenchymal cells (Fig. 3). Optical mapping was used to electrically characterize our sheets, which beat spontaneously at a rate of 78 ± 14 bpm (n = 10) dictated by a singular spontaneous pacing site. The (optical) APD90 was 206 ± 73 ms, which was remarkably similar to the APD90 recorded in spontaneously firing single isolated CMs (247 ± 37 ms). The mean conduction velocity (CV) of electrical propagation from the pacemaker was 5.4 ± 1.3 cm/s which is low compared to CVs in adult human atrial myocardium (60–75 cm/s)^30^, as might be expected from the large extent of I_Na_ inactivation. Bipolar external pacing of sheets at increasing rates (from CL of 800 ms to 150 ms) did not alter the optical APD, but did cause progressive slowing (R^2^ = 0.46, p < 0.0001) of the CV (Supplemental Fig. 4).

**Figure 4:**
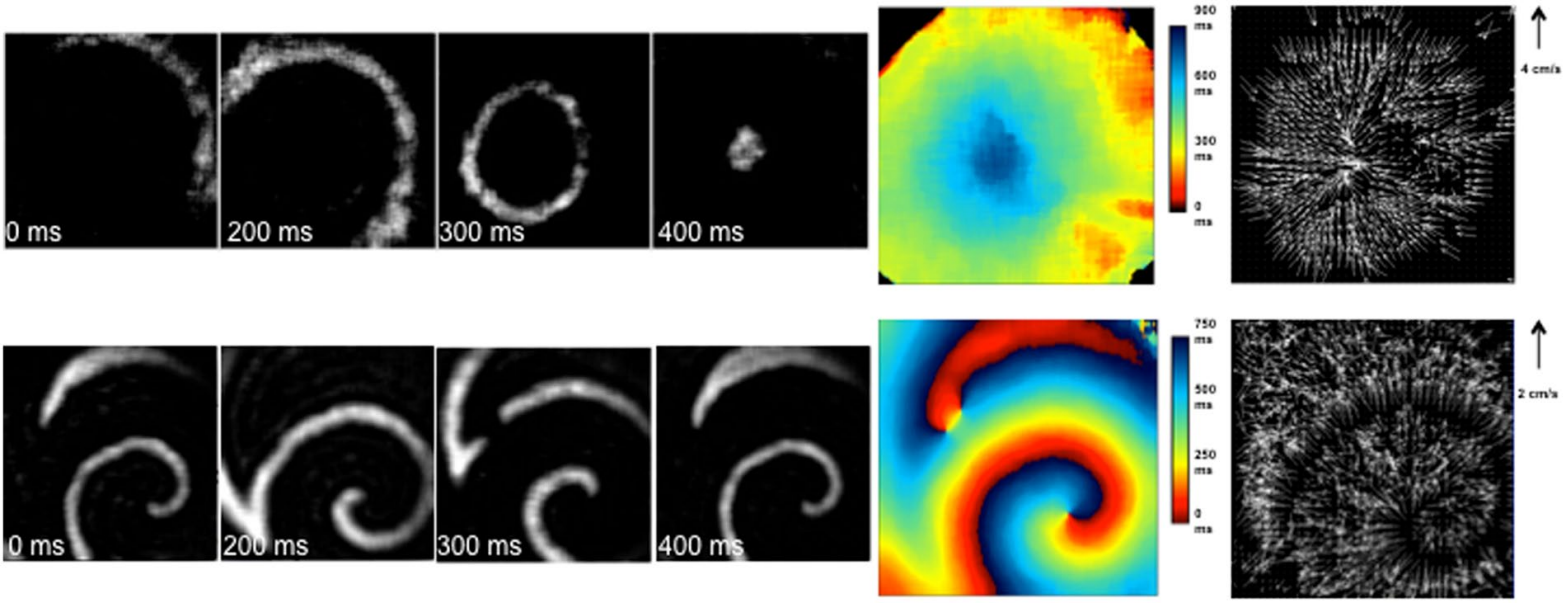
Characterization of atrial CM sheets during spontaneous impulse generation and after rotor induction. Images on the left are still frames, taken every ~100 ms.The top series demonstrates a single focal source starting in the top right hand corner of the image and propagating towards the center. The bottom set of images display a rotor, rotating around an anchor point, which corresponds to the area of slowest conduction velocity. On the right, activation maps and conduction velocity maps corresponding to the images represented on the left.

Consistent with the slow CV, re-entrant rotor patterns of activation were relatively easy to induce (see Methods) and were stable. Figure 4 shows a typical activation pattern (i.e. an activation map) after rotor induction with properties similar to those described in human and animal models with AF^14–16^. The average CL of rotors was 312 ± 30 ms, which was shorter (p = 0.0001) than in spontaneous beatings sheets. Following the induction of rotors, the CV was 3.0 ± 0.9 cm/s which is lower than the CV observed during sinus rhythm (p = 0.0001), as is expected from the source-sink requirements for rotor formation and stability^31^. In addition, the curvature of the rotors (κ, measured within 5 mm of the rotor’s core, was 0.080+/−0.003 mm^−1^ consistent with rotor conformations reported in sheep atria during AF^32^. Further analysis of the activation patterns revealed, as expected^16^, the CVs around the rotor’s core were slower (p < 0.0001) than those measured 50 mm from the core (0.96 ± 0.2 cm/s vs 4.2 ± 0.9 cm/s, Fig. 4). Having established re-entrant rotor activity in our cell sheets, resembling that seen in AF patients, we next explored the effects of dofetilide and flecainide.

### The effect of flecainide in isolated atrial-like CMs and cell sheets

**Figure 5:**
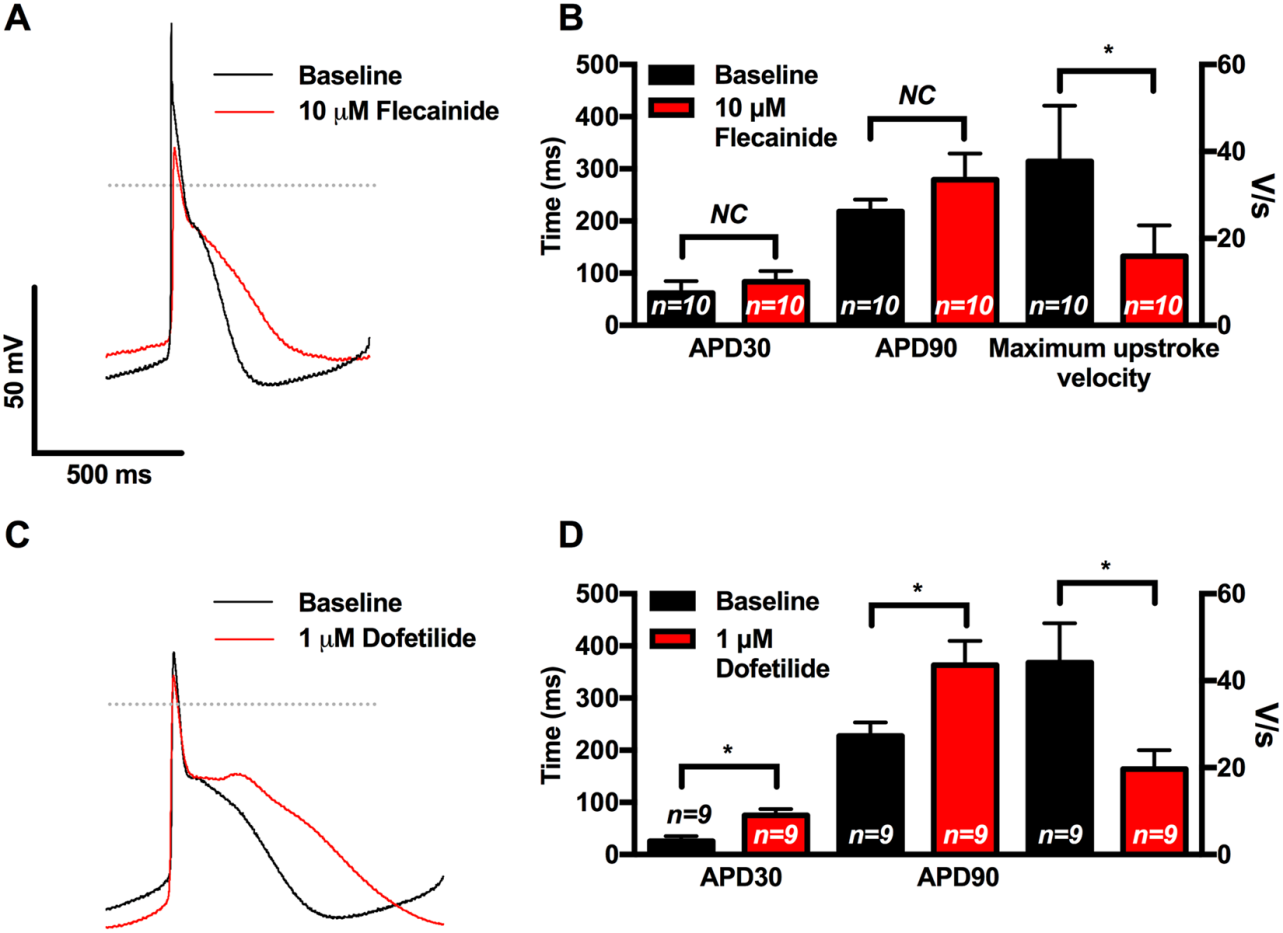
Single cell patch clamp studies demonstrating the effects of flecainide and dofetilide on hESC derived atrial-like CMs. (**A**) Representative APs during spontaneous firing recorded at baseline and with 10 μM of flecainide demonstrating flecainide-mediated slowing of AP upstroke velocity and prolongation of repolarization. (**B**) Compared to baseline APs, flecainide resulted in trends towards prolongation of APD30 and APD90 and slowing of the maximum upstroke velocity (n = 10) (**C**) Representative APs during spontaneous firing recorded at baseline and with 1 μM of dofetilide demonstrating dofetilide-mediated prolongation of repolarization. (**D**) Compared to baseline APs, dofetilide resulted in prolongation of APD30 and APD90 and slowing of the maximum upstroke velocity (n = 9). APD30 = action potential duration at 30% repolarization, APD90 = action potential duration at 90% repolarization, dV/dt_max_ = Maximum action potential upstroke velocity NC = No significant change. *p < 0.05, plots show mean ± SEM.

As expected from its Na^+^ channel blocking properties^33^ as well as I_Na_ measurements under AP-clamp conditions, flecainide (10 μM) treatment of isolated atrial-like CMs reduced (p < 0.001) the maximum upstroke velocities (from 37.8 ± 12.8 to 15.9 ± 7.1 mV/ms) and slowed (p < 0.01) the spontaneous firing rates (CL from 800 ± 70 ms to 1027 ± 94 ms), without affecting the APD (Fig. 5, Supplemental Fig. 5A, Supplemental Table 2). Consistent with slowing of AP maximum upstroke velocity, flecainide reduced I_Na_ in a use dependent manner with the degree of block increasing at shorter CLs (50.6 ± 1.8% block at CL of 2 seconds versus 85.8 ± 2.1% block at CL of 330 ms) (Supplemental Fig. 6). “The absence of changes in APD seems inconsistent with some previous studies demonstrating that flecainide can block late Na^+^ currents. We considered that the absence of APD changes might originate from effects of flecainide on CL. Indeed, regression analyses revealed the expected dependence of APD50 on CL (r^2^ = 0.69, p = 0.0028, slope = 0.30 ± 0.05, n = 10), as seen in human myocardium, in our flecainide treated cells (data not shown). These regression results predict a 69 ms increase in APD50 with CL changes following flecainide. But the increase in mean APD50 was only 34 ms (from 137 ± 26 ms to 171 ± 40 ms with flecainide), suggesting that CL changes may have masked direct actions of flecainide on APD.”

**Figure 6:**
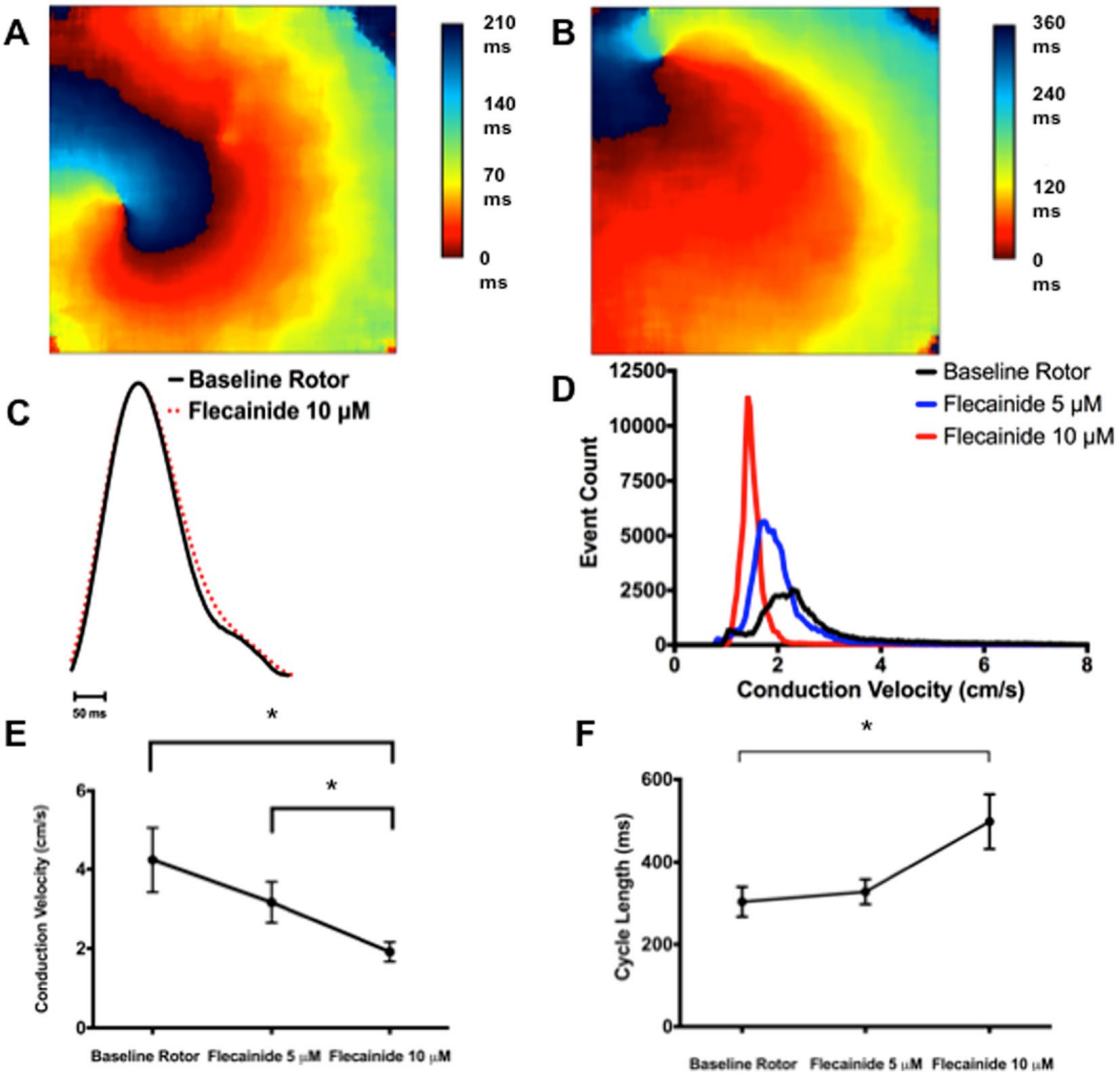
The effects of flecainide on rotor dynamics, APD, CV and CL in atrial-CM sheets. (**A**) and (**B**) Show typical activation maps of an atrial-CM sheet in the absence (**A**) and presence (**B**) of 10 μM flecainide. Notice the spreading of the activation times when flecainide is present indicating a slowing of the CV. C) shows AP traces, average over the entire sheet) in the absence and presence of 10 μM flecainide. Flecainide did not cause APD prolongation. (**D**) Presents a histogram of CVs measured at all points in a CM sheet prior to and after the administration of 5 or 10 μM flecainide. Flecainide shifted the histogram distributions of CVs to lower values as a function of drug concentration. (**E**) Summarizes the CV in 7atrial-CM sheets as a function of flecainide. (**F**) Shows a summary of the CL for the same sheets and conditions shown Panel (**E**). APD = action potential duration, CV = conduction velocity, CL = cycle length. *p < 0.05.

As expected from our isolated CM recordings, flecainide (10 μM) reduced (p = 0.01, n = 7) the CV from 4.1 ± 0.8 cm/s to 2.2 ± 0.4 cm/s at sites distant from the rotor’s core (i.e. >50 mm) without strongly influencing the CV near the rotor’s core (i.e. <5 mm) which is a region known to be electrotonically depolarized^16^ (Supplemental Fig. 7). These effects of flecainide on CV occurred without changes (p = 0.32) in the optically measured APD90 of rotors (142 ± 15 ms vs 125 ± 17 ms, n = 7) (Fig. 6), consistent with previous goat studies^34^. Flecainide also increased (p = 0.03) the rotor’s CL from 304 ± 35 ms to 498 ± 66 ms (Fig. 6) in conjunction with reductions (P < 0.01) in κ in the rotor’s core by a factor of 1.11+/−0.04 (from ~0.083 to ~0.075 mm^−1^). Although these actions of flecainide are consistent with previous animal, human and modeling studies establishing that Na^+^ channel blockade terminates and prevents AF^34–36^, flecainide did not terminate rotors in our cells sheets (See Discussion).

### The effects of dofetilide on the isolated atrial CMs and cell sheets

As expected from its specific HERG-blocking properties, we found that dofetilide (1 µM) prolonged (n = 9, p < 0.01) APD90 in isolated atrial-like CMs consistent with the presence of dofetilide-sensitive tail currents (3.96 ± 1.39 pA/pF, n = 6) in voltage-clamp studies designed to detect the presence of HERG-currents^37^ (Fig. 5 and Supplemental Table 3). Dofetilide also reduced the AP upstroke velocity (p < 0.05) and demonstrated a trend towards slowing (p = 0.06) of the spontaneous firing rates, which, as with flecainide, is expected to affect APD independently of direct HERG inhibition. (Supplemental Table 3, Supplemental Fig. 5).

**Figure 7:**
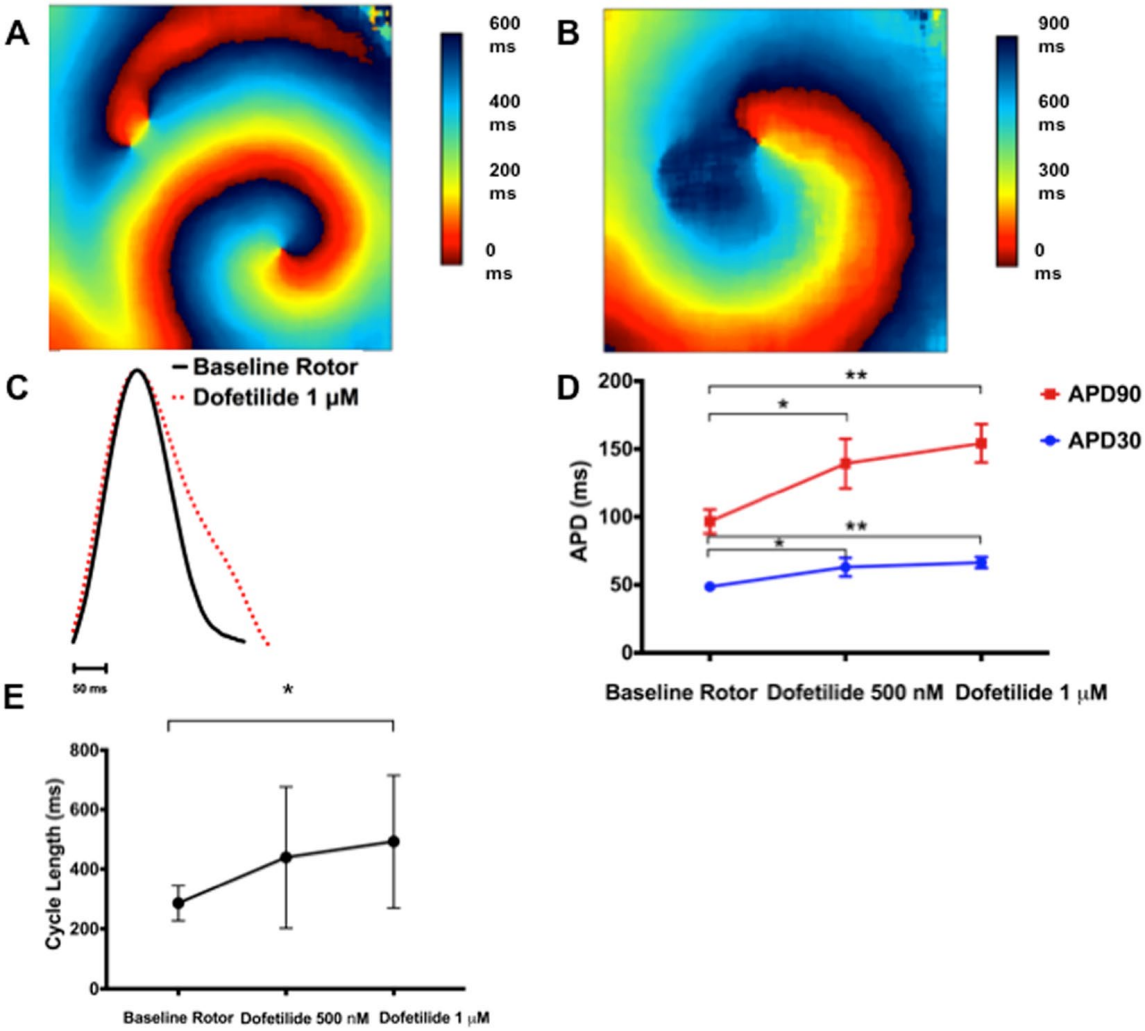
The effects of dofetilide on rotor dynamics, APD, and CL in atrial-CM sheets. (**A**) and (**B**) Show typical activation maps of an atrial-CM sheet in the absence (**A**) and presence (**B**) of 1 μM dofetilide. In this case there is, as with flecainide, a spreading of the activation times consistent with slowing of the CV. (**C**) Shows AP traces, average over the entire sheet, in the absence and presence of 1 μM dofetilide. Clearly, dofetilide caused notable APD prolongation. (**D**) Summarizes the APD30 and APD90 for 8 atrial-CM sheets. The data shows that APDs are prolonged following the addition of both 500 nM and 1 μM dofetilide. E demonstrates slowing of the CL in the atrial-CM sheets with increasing doses of dofetilide (n = 8). APD = action potential duration, CV = conduction velocity, CL = cycle length.*p < 0.05, **p < 0.01.

In our cells sheets, Fig. 7 shows that dofetilide prolonged (p < 0.01, n = 8) the optical APD (APD90 from 97 ± 9 ms to 154 ± 14 ms) and also slowed (p = 0.03) CL (from 285+/− 59 ms to 492+/− 223 ms) without measurably affecting either CV or κ consistent previous modeling results as well as human and animal studies^38, 39^ suggesting that HERG currents can influence the source currents near the core of electrical rotors. Moreover, dofetilide was able to induce cardioversion in 1 of 10 sheets, consistent with the physical expansion of the reentrant structures and cardioversion seen in sheep with persistent AF^34^ (See Discussion).

## Discussion

We have generated atrial-like CMs through a directed differentiation protocol to enrich for atrial-specific molecular markers (NPPA, KCNJ3, GJA5) versus ventricular-specific markers (MYL2, IRX4), that have predominantly atrial-like electrophysiologic properties^11^. Multicellular sheets of atrial-like CMs displayed electrical wavefront activation establishing electrotonic coupling and regenerative AP propagation during spontaneous depolarizations and following external electrical pacing. The optical AP profiles were remarkably similar to those measured in our isolated CMs and to those seen in adult human atrial preparations^11^. CVs in cell sheets (ranging from 2.5 to 8.5 cm/s) were similar to CVs reported in (presumably) ventricular sheets cultured for similar periods^40, 41^ but much slower than reported in human atria^30^. While several factors could contribute to this slow CV (i.e. gap junction channel densities and CM connectivity^42^), a major factor is likely the relatively positive resting diastolic membrane potential which promotes sodium channel inactivation^43^. Indeed, ~60% of the Na^+^ current was inactivated in our CMs as a consequence of diastolic depolarization. Moreover, when pacing rates were increased, further CV slowing was observed along with the appearance of APD alternans preceding rotor formation (Supplemental Fig. 8), both of which are predicted to promote arrhythmias^44–46^. This paper goes on to characterize and interrogate the first hPSC derived model of atrial fibrillation.

Recent clinical studies have provided evidence for the presence of re-entrant spiral waves (i.e. rotors) which degenerate into fibrillatory atrial activity in AF^14^ and which can potentially be targeted for ablation^15^. This work draws from, and supports, the findings generated by *in vivo* contact mapping and optical mapping of AF demonstrating self-organized and self-sustaining drivers^47–50^. Rotors are functionally re-entrant rhythms rotating around an unexcited core, or phase singularity (PS) and rely critically on the atrial wavelength (i.e. λ), which is the product of the ERP (~APD), and the CV^51^. The re-entrant rhythm also relies on an excitable gap, or tissue in the circuit that has complete recovery of excitability and can be reactivated by the next electrical wavefront^52^. Rapid pacing of our multicellular sheets induced clear rotor geometry around a PS from which activation wave fronts emulated with progressive reductions in curvature, and faster CVs as the distance from the PS increases, precisely as expected from previous simulation and animal studies^16^. In addition, the CLs of rotors were about 3-fold faster than the intrinsic CLs in sinus rhythm. These observations support the conclusion that our rotors can provide important insights into the mechanisms of action of AADs on the treatment of AF. Importantly, our optical mapping provides significantly higher resolution of rotors, compared to any tools that are clinically available.

We first examined the effects of flecainide, a commonly used class I antiarrhythmic agent, which blocks sodium current (I_Na_)^33^ as well as selected potassium channels (i.e. especially I_Kr_) in animal models^35, 53^. While the mechanism of action of this agent has previously been studied in animal models and computer simulations, no previous studies have directly explored its effects on rotor properties in human myocardium. Moreover, previous studies of flecainide’s effects on AF in animal models are conflicting, with one study concluding that flecainide’s antiarrhythmic effect does not require prolongation of the effective refractory period (ERP)^54^ while in another, APD prolongation correlated more strongly to AF termination than I_Na_ blockade^35^. Computer simulations, contrived to mimic native human atrial myocardium, have concluded that the actions of flecainide and pure Na^+^ channel blockade on “fibrillating” tissues are best understood in terms of rotor dynamics^44, 55, 56^. Accordingly, our studies demonstrate, for the first time in isolated human atrial tissue, that flecainide alters rotor properties in a manner entirely consistent with its actions in clinical AF. Specifically, flecainide had no effect on APD, but profoundly slowed the CL and reduced the curvature (in the inner regions) of rotors. These observations are consistent with flecainide’s effects in our isolated CMs, wherein the drug impaired maximum upstroke velocities of APs and rate-dependent reductions in I_Na_, without affecting APD. While the absence of APD changes is somewhat unexpected since flecainide is expected shorten the APD by blocking late I_Na_, CL slowing combined with I_Kr_ blockade by flecainide may have masked the effects I_Na_ blockade^57^. Consistent with this, APD (but not upstroke velocity) depends strongly on AP shape in human atrial fibers, suggesting that flecainide’s effects on APD may be complicated by variable channel expression^58^.

The changes in our rotor properties with flecainide are readily explained by I_Na_ reductions which limit the source currents needed to maintain rapid rotor activation and tight rotor curvatures^34, 59^. Consequently, the termination and prevention of AF by flecainide, as well as other antiarrhythmics, is associated with slowed CLs seen with surface ECG traces as well as catheter recordings in patients and animals^60–62^. These changes induced by flecainide have been linked to increased organization as measured by spatial and temporal correlation between different electrical wavefront, which appear necessary for successful cardioversion with both Class I and III antiarrhythmics in goats with persistent AF^60^. Similar features were clearly observed in our sheets. Indeed, flecainide increased the estimated rotor size (determined by CV*CL) from 1.1 cm to 1.9 cm as well as the estimated spatial excitability gap from 0.66 to 0.82 cm (estimated as CV*(CL-APD90)(Direct visual evidence for this increase in rotor dimensions is provided in Supplemental Fig. 9). Rotor expansion and reductions in rotor curvature are also predicted to enhance meandering of the rotor’s core leading to rotor destabilization and promoting extinction^63–65^. Although core meandering was not assessed in our studies due to limited recording times, flecainide was unable to cardiovert rotors, suggesting that core meandering was probably limited in our sheets. This observation is consistent with the relatively low CVs seen in our atrial sheets, even in the absence of drug, as discussed above.

Previous studies have established that cardioversion with Class III antiarrhythmics is associated with slowing of CLs and increased temporal and spatial electrical organization during AF^34, 61^. This is consistent with increased tissue wavelengths resulting from APD prolongation related to selective inhibition of the rapid delayed rectifier potassium current (I_kr_)^66^. Despite these observations, as a result of rapid atrial remodeling associated with rapid firing of atria, Class III antiarrhythmics targeting I_kr_ can have modest effects on the ERP, suggesting that AF cardioversion is not solely a consequence of APD prolongation, and modeling studies disagree on the extent to which I_Kr_ effects rotor core dynamics^67–69^. Our studies show that dofetilide causes APD prolongation in isolated atrial-like CMs and atrial-like cells sheets, consistent with the presence of dofetilide-sensitive currents. These actions were associated in cell sheets with marked slowing of the CL, which also trended towards prolongation in isolated CMs. In addition, both and CV in rotors tended to be reduced, as predicted in previous modeling studies by Qu *et al*.^68^. These trends are similar to the changes in CV and CL seen with flecainide and could arise from reductions in I_Na_ as a consequence of depolarization of the diastolic membrane potentials (data not shown), due to the reduced slowly deactivating I_K,r_ as reported previously^70^ which also explains the reduced AP upstroke velocities seen with dofetilide in our isolated cardiomyocytes. These observations suggest that Class I and Class III antiarrhythmic actions may originate from common shared mechanisms, at least under our experimental conditions. Consistent with this suggestion, we found that dofetilide, like flecainide, increased the temporal excitability gap from 377 ms to 662 ms (estimated as the CL-APD90), and an increase in the excitability gap was the only parameter linked previously to cardioversion for both Class I and III antiarrhythmic agents in goats with persistent AF^34^. Despite the effects of dofetilide on APD, we did not, however, observe head-to-tail infringement, consistent with small expansion of the spatial excitability gap in our sheets.

## Conclusion

In this study, we have generated, for the first time, rotors in atrial-like cell sheets derived from hPSCs that carry many of the characteristics of rotors that drive human AF. Although our cell sheets suffered from slow CV associated with depolarized diastolic potentials which characterize immature myocardium, the actions of flecainide and dofetilide, in our cell sheets are remarkably in line with current rotor theory, and consistent with known actions of these agents in AF seen in animal models and humans. Importantly, although the two agents had distinct actions on rotor parameters, both agents expanded the temporal excitability gap suggesting overlapping antiarrhythmic mechanisms. Thus, while the translational potential of our platform might be limited by developmental immaturity, hESC-CM are expected to have a profound impact on precision AAD use in AF, particularly with future developments in myocardial maturation and genomic editing.

## Electronic supplementary material

Supplementary Material
