# Supplementary material for: Modeling Atrial Fibrillation using Human Embryonic Stem Cell-Derived Atrial Tissue
Source: Sci Rep. 2017 Jul 13;7:5268. doi: 10.1038/s41598-017-05652-y (PMC5509676; doi:10.1038/s41598-017-05652-y)
Supplement: Supplementary file 1 — Supplementary Material [file 41598_2017_5652_MOESM1_ESM.doc]

Supplement

Modeling Atrial Fibrillation using Human Embryonic Stem Cell-Derived Atrial Tissue

Zachary Laksman M.D.^1*^, Marianne Wauchop B.Sc.^2^, Eric Lin Ph.D.^3^, Stephanie Protze Ph.D.^4^, Jeehoon Lee B.Sc.^5^, Wallace Yang P.Eng.^6^, Farzad Izaddoustdar M.Sc.^8^, Sanam Shafaattalab B.Sc.^9^, Lior Gepstein M.D., Ph.D.^10^, Glen F Tibbits Ph.D.^11^, Gordon Keller Ph.D.^12^, Peter H Backx Ph.D.^13^

Supplemental Methods

Culture of hESCs and directed differentiation of atrial- and ventricular- like cardiomyocytes

HES3 NKX2-5^egfp/w^ cells were maintained on irradiated mouse embryonic feeder cells in hESC media consisting of DMEM/F12 (50:50;MEdiatech, Herndon, VA) supplemented with 20% knock-out serum replacement (SR), 100 μM nonessential amino acids, 2mM glutamine, 50U/mL penicillin, 50 μg/ml streptomycin (Invitrogen Grand Island, NY), 10^-4^ M Β-mercaptoethanol (Sigma, St Louis, MO), and 20 ng/mL hbFGF (R&D Systems, Minneapolis, MN) in 6-well tissue culture plates ^1^. The HES3 NKX2-5^egfp/w^ cell line expressed cell surface makers that characterize undifferentiated human cells ^2^ (Supplemental Figure 2). Embryoid bodies for differentiation were generated after feeder depletion and growth of ES cell colonies to 80% confluence. The aggregates were generated at 37°C in a hypoxic environment of 5% CO2, 5% O2, and 90% N2 on a rotator set at 70 rpm for 24 hours, were then spun in a centrifuge at 300 rpm, washed and then re-suspended in the induction media. We employed a cocktail of cytokines for the directed differentiation of hESCs into cardiomyocytes using established differentiation protocols that employ Activin A and BMP4 signaling for mesoderm induction followed by Wnt inhibition for cardiac specification (Supplemental Figure 1)^3, 4^. To drive cardiomyocytes towards an atrial phenotype, we added retinoic acid (RA) after mesoderm formation, as discussed previously ^5, 6^.

Flow cytometry and cell sorting

For cell-surface antigens, staining was carried out in PBS with 3% FCS. For intracellular antigens, staining was carried out on cells fixed with 4% paraformaldehyde in PBS. Staining was done in PBS with 3% FCS and 0.5% saponin (Sigma). Cells were stained at a concentration of 2.5 × 10^6^ cells/ml with anti-CD56-APC (BD Pharmigen; 1:100) and anti-PDGFRA– PE (R&D Systems; 1:20), anti-SIRPA–PE-Cy7 (clone SE5A5; BioLegend; 1:500), anti-CD90-APC (BD Pharmingen, 1:2000) anti-CTNT (clone 13-11; Thermo NeoMarkers; 1:2000), goat anti-mouse IgG–APC(BD; 1:200). Stained cells were analyzed on an LSRII flow cytometer (BD Biosciences). For FACS, the cells were sorted at a concentration of 10^6^ cells/ml in IMDM/5% FCS using a FACSAriaTMII (BD Biosciences). For the sorting strategy, Cardiac Troponin (cTNT), NKX2.5 (expressed early in atrial cells in the cardiac mesoderm stage of development) and the surface marker SIRPA, which has previously been shown to uniquely mark the cardiomyocyte lineage in hPSC differentiation cultures, were used to determine differentiation efficiency and as positive markers of cardiomyocytes^3, 4, 7^. CD90+ cells were removed during sorting to exclude ill defined contaminant cells that may include fibroblasts. Data were analyzed using FlowJo software (Treestar).

Quantitative Real-Time PCR

Total RNA was prepared with the RNAqueous-micro Kit (Ambion) followed by a DNase

digestion step (Ambion). 100 ng to 1 μg RNA was reverse

transcribed into cDNA via random hexamers and Oligo (dT) with Superscript

III Reverse Transcriptase (Invitrogen). Real-time quantitative PCR was performed

on a MasterCycler EP RealPlex (Eppendorf). All experiments were done using the QuntiFast SYBR Green PCR Kit (Qiagen) according to the manufacturer’s instructions. Primers were purchased from Integrated DNA Technologies(Supplemental Table ). A 10-fold dilution series of sonicated human genomic DNA standards ranging from 50 ng/ml to 5 pg/ml was used to evaluate the efficiency of the PCR and calculate the copy number of each gene relative to the housekeeping gene TBP.

Immunostaining and Microscopy

Immunostaining was performed as previously described ^7^ using rabbit anti-cTNI (Abcam; 1:100) and mouse anti-Vimentin (Sigma-Aldrich, 1:100). Secondary antibody used were donkey anti-mouse IgG-Alexa 488 (Invitrogen; 1:300) and donkey anti-rabbit IgGCy3 (Jackson ImmunoResearch; 1:300). DAPI (Life Technologies, SlowFade Gold) was used to counterstain nuclei. The stained imaged were captured using a confocal microscopy system (Yokogawa CSU-X1,Calgary, AB, Canada) with a Quantem 512c EMCCD camera (Photometrics, Tucson, AZ, U.S.A) and analyzed using Image-Pro Plus (Media Cybernetics Inc., USA).

Single Cell Electrophysiology

Spin EBs resulting from control and RA-treated differentiations were dissociated at day 20 to single cells using type 2 collagenase (Worthington biochemical corporation) and TrypLE (Thermo fisher). Cells were sorted based on DAPI- /SIRPA+/GFP+/CD90- surface markers as previously described to generate a pure population of CMs. Single cells were plated on 15 to 25 percent matrigel-coated coverslips. Electrophysiological measurements were performed 7-10 days after dissociation, sorting and plating.

Pipettes were positioned with a micromanipulator (Burleigh PCS-250 system) mounted on the stage of an inverted microscope (Olympus IX70). Membrane potential was controlled with an Axon headstage (CV 203BU) connected to an Axopatch 200B voltage- clamp amplifier (Axon Instruments, Foster City, CA). Data was digitized (Axon Digidata 1322A) and acquired using Axon Clampex software (pClamp version 8.2.0.235/9.2.1.9). An agar-salt bridge was used as the reference electrode. Data was analyzed using Clampfit (Molecular Devices, Sunnyvale, CA, U.S.A).

Whole-cell patch clamp recordings were used to characterize the electrophysiological properties of single cells. To prevent dialysis of the cells and to maintain more physiologically relevant intracellular conditions than ruptured patch, perforated patch clamping was employed using nystatin (Sigma-Aldrich, N3503) as a perforating agent for all experiments except sodium current recordings in which cell rupture was used. AP and current recording were performed at room temperature (22-23 °C). Pipettes were pulled from borosilicate glass (with filament 1.5 mm OD, 0.75 mm ID, Sutter Instrument Company) using a Flaming/Brown pipette puller (model p-87, Sutter Instrument Company) and were heat polished. Myocytes were placed into and perfused with a bath solution of tyrodes containing (in mmol/L): 140 NaCl, 4 KCl, 1.2 CaCl_2_, 1 MgCl_2_. 15 NaHCO_3_, 10 HEPES and 10 D-glucose (pH 7.35 with NaOH). For sodium current recordings, following gigaseal formation and membrane rupture in tyrodes, cells were perfused with solution containing (in mmol/L) 140 TEA-Cl, 10 NaCl, 4 KCl, 1 MgCl_2_, 10 HEPES, 10 glucose, 0.005 nifedipine and 0.01 NiCl_2_ (pH 7.35 with NaOH) through PE90 tubing mounted on a manipulator and brought close to the cell. To examine the effects on AP and current profiles, dofetilide (500 nM and 1 μM; Sigma-Aldrich, PZ0016) and flecainide (10 μM; Sigma-Aldrich, P6777) were added to the bath solution. The pipette resistance ranged from 3 to 6 MΩ when filled with internal solution containing (in mmol/L): 100 K aspartate, 20 KCl, 5 NaCl, 1 MgCl_2_, 5 MgATP and 10 HEPES (pH 7.2 with KOH) for AP and potassium current recordings and (in mmol/L): 135 CsCl, 10 NaCl, 1 CaCl_2_, 10 EGTA, 1 MgCl_2_, 4 MgATP and 10 HEPES (pH 7.2 with CsOH) for sodium current recordings. Nystatin stock was prepared daily in DMSO by vortexing and 15 seconds of ultrasonication at 22 kHz to a final concentration of 30 mg/ml. To maintain potency, nystatin stock was mixed with internal solution every hour by vortexing for 60 seconds to a final concentration of 200-350 μg/ml (the exact concentration was determined based on the condition of the cell membranes). All nystatin-containing solutions were protected from light and kept on ice. Pipette tips were prefilled by briefly (<1 sec) dipping the blunt end in nystatin-free internal solution before immediately backfilling with nystatin-containing solution using a syringe. To prevent nystatin from interfering with gigaseal formation, gigaseals were formed within 1 minute of pipette filling. Nystatin adequately perforated the membrane within 5 to 30 minutes of gigaseal formation. AP and current recordings were initiated once access resistance decreased below 50 and 30 MΩ respectively.

Single cell studies were performed in current- and voltage-clamp configurations to record APs during spontaneous firing and following anode breaks (the injection of negative current for 1 second to hold cells at -80 to -90 mV) and ionic currents respectively. Voltage-gated potassium currents were measured using a 500 ms step from a holding potential of -80 mV to +60 mV followed by a 2000 ms step back to -40 mV. The voltage-steps to +60 mV were chosen to reduce the contributions of inward Ca^2+^ and Na^+^ currents. The steps back to -40mV were used to quantify the tail currents generated by human ether-a-go-go related (HERG) channels.  Voltage-gated sodium currents were measured using 50 ms steps from a holding potential of -100 mV to -30 mV at 0.5, 1 and 3 Hz. For current recordings, the cell capacitance was determined by integrating the area under the capacitance transient in response to 10 mV steps and was used to calculate current density. Cell capacitance and series resistance were compensated by 60 percent.

Preparation of cell sheets

150 μL of 100% concentrated matrigel drops were placed in the centre of 35 mm tissue culture treated petri dishes (Falcon). The dishes were kept on ice for 30 minutes and then the matrigel was removed leaving a thin coat of matrigel with a diameter of 1 cm. The petri dishes were incubated at 37 °C overnight. hESC-CMs were digested using collagenase B at a concentration of 250 U/ml in Hank’s balanced solution (Life technologies) overnight at a concentration of 5 million cells/ml. TrypLE was used to make single cell suspensions which were plated on the matrigel coated petri dishes at a concentration of 1.5 million cells per 150 μL drop in backbone media plus 1 ng/ml ROCK inhibitor (Y-27632 Dihydrochloride Hydrate,Toronto Research Chemicals, cat.no Y100500) generating cell sheets of 1 cm diameter.

Optical Mapping

Imaging was performed in IMDM supplemented with NaCl (final concentrations in mmol/L: 140 NaCl, 3.6 KCL, 1.2 CaCl2, 1 MgCl2. 10 HEPES and 5.5 D-glucose). The petri dish was placed on a plate warmer and maintained at temperature of 36-37°C. The tissue was illuminated using a mercury light source (X-Cite Exacte, Lumen Dynamics, Mississauga, ON, Canada) with a 525 ± 50 nm band-pass filter. Fluorescent light was collected using a 645 ± 75 nm band-pass filter. Images were captured using an electron multiplying charge coupled device (EMCCD) camera (Cascade 128+, Cascade Evolve, Photometric, Tucson, AZ, U.S.A) connected to an Olympus MVX-10 upright microscope (Center Valley, PA, U.S.A) equipped with a 0.63x c-mount adapter and a 0.38x lens relay. Frames were captured at 522 fps at 1X1 binning, using Image Pro Plus (Media Cybernetics, Rockville, MD, U.S.A) software.

Electrical Stimulation (Pacing) and Rotor Induction

Stimulation and sensing electrodes were constructed from platinum wire coated with Sylgard for electrical insulation. Stimulation electrodes were designed in a bipolar configuration in order to achieve point stimulation. These electrodes were connected to Pulsar 6i & 6b stimulators (FHC Inc, Bowdoinham, ME, U.S.A) for programmed stimulation. Capture threshold was by simultaneous optical mapping of signals to confirm rate and was achieved using 40mV with a pulse duration of 2 ms. The bipolar electrodes were able to pace and generate point stimulation wavefronts which propagated through the cell sheet. Restitution curves were generated by pacing at a fixed cycle length for 30 seconds prior to recording. Acquisition times varied between 5 and 15 seconds in length at a time, with multiple sequential recordings performed on the same tissue. Acquisition was performed either during the time of electrical stimulation during the generation of restitution curves, or directly after stimulation for the recording of rotor activity. Thus we were directly looking at the acute onset of atrial fibrillation without the potentially confounding effects related to atrial fibrillation progression simulating clinically paroxysmal atrial fibrillation compared to persistent or chronic atrial fibrillation. Rotors were induced either during restitution testing (median cycle length (CL) = 200 ms, range 150-300 ms) or burst pacing (CL of 50 ms for 30 seconds). Burst pacing uniformly induced rotor activity.

Signal Processing

Signal processing of the optically mapped data was performed using a custom IDL software program or Scroll software (courtesy of Sergey Mironov). For each image pixel, the signal intensity from the surrounding ±3 pixels was averaged to allow for individual cardiac cycles to be identified as described^8^. In brief, baseline intensities were normalized by fitting the data to a polynomial function and the data intensity normalized to 0->1. To reduce high frequency noise, a butterworth filter was applied using a frequency cut-off set at 30 Hz. Individual cycles were then segmented by applying a threshold value of 50% and the peak of each AP was used for the activation time of a given cardiac cycle for that location. For action potential duration (APD) measurements, the signal-to-noise ratio was further increased by averaging multiple cardiac cycles for a given pixel location.

To calculate instantaneous conduction velocities, activation times were fit to the equation of a plane (**a**x +**b**y + cz = d), in which **a** and **b** reflect the x and y components of the conduction velocity. Instantaneous conduction velocities were used to generate vector maps. The report of conduction velocities used average conduction speeds instead of instantaneous because taking spatial averages from instantaneous conduction velocities overestimates the velocities (it can be mathematically proven). Average conduction speeds were calculated by tracing the path of every pixel of wave fronts throughout the entire cardiac cycle and dividing the path length by time.

Curvature is the rate of change in the tangential unit vector along a contour with respect to contour length. It can be shown that a circle with radius r has a curvature of 1/r. Thus a line has a curvature of 0. Curvature maps were generated by first taking activation maps and finding gradients at all pixels. The gradients were rotated by 90 degrees and normalized to obtain tangential unit vectors. For each pixel, the tangential unit vector was expanded out to a circle of 4 pixel radius at p1 and p2, and the difference of the tangential unit vectors were used to calculate curvature.

Supplemental Results

Supplemental Table 1: Quantitative Real Time PCR primers purchased from Integrated DNA Technologies

| Primer name | Forward primer 5'-3' | Reverse Primer 5'-3' |
| --- | --- | --- |
| NPPA | GGGTCTCTGCTGCATTTGTGTCAT | AGAGGCGAGGAAGTCACCATCAAA |
| KCNJ3 | TCATCAAGATGTCCCAGCCCAAGA | CACCCGGAACATAAGCGTGAGTTT |
| GJA5 (Cx40) | aatcttcctgaccaccctgcatgt | cagccacagccagcataaagacaa |
| Myl2 | TGTCCCTACCTTGTCTGTTAGCCA | ATTGGAACATGGCCTCTGGATGGA |
| Irx4 | TCATCAAGATGTCCCAGCCCAAGA | CACCCGGAACATAAGCGTGAGTTT |

Supplemental Table 2: The effects of 10 μM flecainide on AP parameters in single atrial-like cells recorded during spontaneous firing.

|  | N  (cells) | dV/dt_max_  (V/s) | DMP  (mV) | CL  (ms) | APD30 (ms) | APD90 (ms) |
| --- | --- | --- | --- | --- | --- | --- |
| Baseline | 10 | 37.75 ± 12.77 | -57.91 ± 2.08 | 799.67 ± 70.26 | 62.06 ± 23.00 | 218.40 ± 23.07 |
| 10 μM Flecainide | 10 | 15.92 ± 7.08* | -52.16 ± 3.00 | 1027.32 ± 94.11* | 83.72 ± 20.27 | 279.47 ± 49.79 |

dV/dt_max_ was also slowed (p<0.0001, n=14) when APs were evoked following membrane hyperpolarization to -85mV (anode break) from a baseline of 76.99 ± 13.78 V/s to 36.33 ± 8.82 V/s following flecainide (data not shown). dV/dt_max_ = maximum upstroke velocity, DMP = diastolic membrane potential, CL= cycle length, APD30 = action potential duration at 30% repolarization, APD90 = action potential duration at 90% repolarization, values for mean ± SEM are shown, *p<0.05)

Supplemental Table 3: The effects of 1 μM dofetilide on AP parameters in single atrial-like cells recorded during spontaneous CM firing.

|  | N  (cells) | dV/dt_max_  (V/s) | DMP  (mV) | CL  (ms) | APD30 (ms) | APD90 (ms) |
| --- | --- | --- | --- | --- | --- | --- |
| Baseline | 8 | 47.98 ± 9.34 | -63.63 ± 4.04 | 724.53 ± 89.67 | 26.11 ± 10.83 | 211.45 ± 23.21 |
| 1 μM Dofetilide | 8 | 20.78 ± 4.82* | -51.37 ± 4.71* | 914.03 ± 189.21 | 80.93 ± 11.89* | 327.04 ± 33.29* |

Note: Consistent with previous observations dofetilide resulted in an upward drift in the DMP from -63.63 ± 4.04 to -51.37 ± 4.71 (n=8, p<0.05). These observations suggest that HERG channels (i.e. I_kr_) is a major determinant of the maximal diastolic membrane potential (DMP) in our atrial CMs, presumably as a consequence of the relatively low I_K1_ expression as reported previously in ventricular CM from hESC-derived^9^. The drift in DMP is expected to lead to reductions in I_Na_ densities. Consistent with this suggestion, dV/dt_max_ was not affected (p=0.1661, n=12) by dofetilide when APs were generated after the introduction of anode breaks designed to hold the membrane potential at -85mV (i.e. dV/dt_max_ was 66.65 ± 11.12 V/s in the absence of dofetilide compared to 56.04 ± 13.12 V/s after the addition of 1 μM Dofetilide, data not shown). dV/dt_max_ = maximum upstroke velocity, DMP = diastolic membrane potential, CL = cycle length, APD30 = action potential duration at 30% repolarization, APD90 = action potential duration at 90% repolarization, values for mean ± SEM are shown, *p<0.05

**Supplemental Figures**

Supplemental Figure 1: Scheme of the protocol used to differentiate hESCs towards the cardiomyocyte lineage highlighting the three main stages of development: 1) mesoderm induction, 2) cardiovascular specification and 3) maturation. Protocols for the generation of ventricular-like and atrial-like cardiomyocytes differed in terms of whether or not retinoic acid (RA) was added to the cultures at after mesoderm formation. ES = embryonic stem cell, RA = retinoic acid.


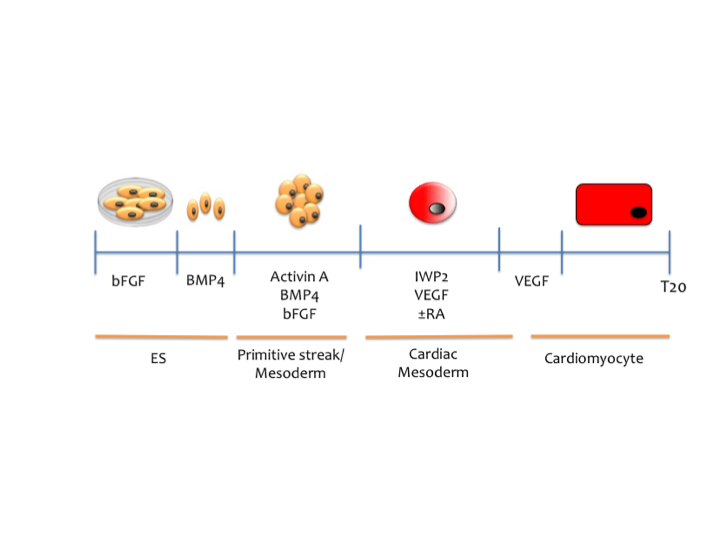


Supplemental Figure 2: Flow cytometric analyses of the frequencies of cell surface markers used routinely to identify undifferentiated human embryonic stem cells (hESCs); stage specific embryonic antigen 3 and 4 (SSEA3, SSEA4) and two human EC cell antigens Tra-1-60 and Tra-181 also used to mark undifferentiated hESCs. SSEA1 is expressed on differentiated hESCs. Flow cytometry performed after 5 days of hESC culture on irradiated mouse embryonic fibroblasts at the time point when cells are dissociated for the T0 of differentiation. Here we show, based on gates set on the unstained sample on the far left, high frequencies of markers of undifferentiated hESCs and a low frequency of a marker of differentiated hESCs.


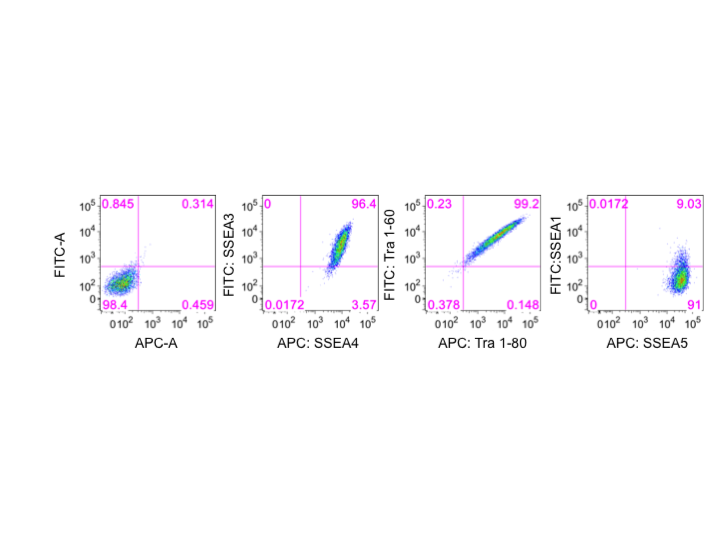


Supplemental Figure 3: Flow cytometric analyses plotting the frequencies of several important markers used in the optimization and validation of the differential protocols employed. The plot on the left demonstrates an optimal profile on day 4 with a high frequency of CD56+ and PdfR-α+ cells. The middle plot is representative of a good differentiation protocol which generates a high frequency of SIRPα+ cells. The frequency of CD90+ cells is also routinely assessed, and employed to optimize purification of cardiomyocytes and to quantify the frequency of CD90+ cells in the cell sheet culture. The final plot on the right demonstrates an efficient differentiation protocol generating 91.9% cTNT+ cells.


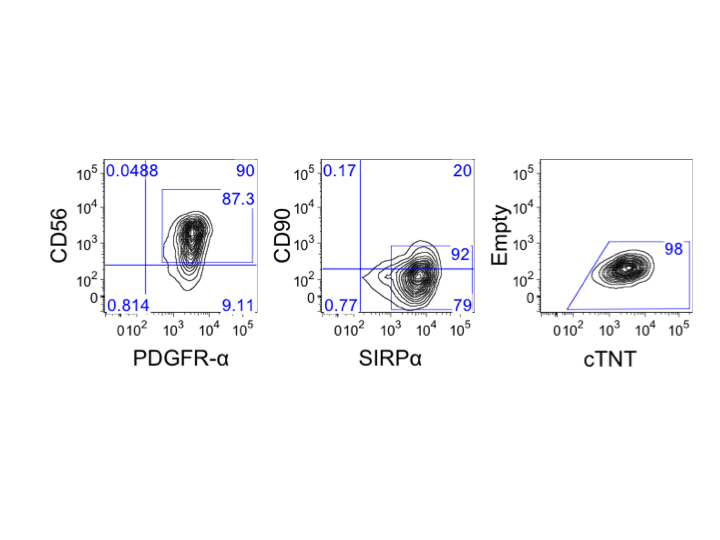


Supplemental Figure 4: Characterization atrial-CM sheets using optical mapping. Cell sheets included in the figure were generated from cardiomyocytes that have undergone the atrial differentiation protocol with the inclusion of retinoic acid. Cell sheets portrayed in this figure underwent pacing protocols, but were not exposed to anti-arrhythmic dugs (n = 5). A. An example of a typical recording of the changes in fluorescence intensity in a localized region of the sheet. The signals were processed to illustrate AP conformation. B. Shows the results of action potential duration estimates at 50% repolarization as a function of the pacing cycle length for 5 atrial-CM sheets. Clearly, the CL has minimal effects on action potential duration of our sheets. C. Summarizes the relationship between pacing CL and conduction velocity (CV) for 5 cell sheets using all data point available. D. Summarizes the relationship between CL and CV for 5 atrial-CM sheets. The results demonstrate a consistent pattern in slowing of conduction velocity at faster rates. APD50 = action potential duration at 50% repolarization.


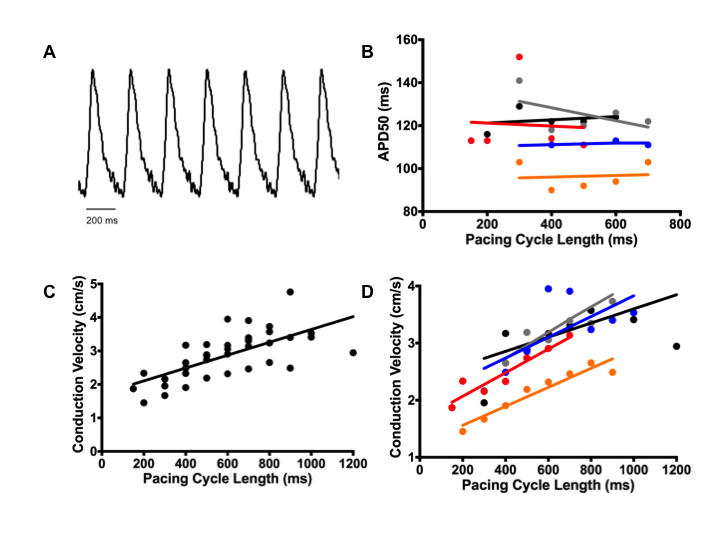


Supplemental Figure 5: Changes in cycle length and APD50 with flecainide and dofetilide in our isolated atrial-like CMs. A. and B. Changes in cycle length, APD50 and percent change from baseline upon addition of 10 μM flecainide and 1 μM dofetilide respectively. Note that for dofetilide a single outlier was identified and removed. * p<0.05 for comparisons to baseline measurements or to zero (for % change), NC= No significant change, APD50=Action potential duration at 50% repolarization.

Supplemental Figure 6: A. Representative baseline sodium current recording generated using the protocol shown with the current generated by multiple sequential pulses superimposed. B. Representative sodium current recording from the same cell and generated using the same protocol as shown in Panel A in the presence of 10 μM flecainide demonstrating use-dependent sodium channel blockade.

C. Quantification of the relative changes in peak sodium current amplitude with different CLs. This demonstrates frequency-dependent flecainide-mediated blockade characterized by faster and greater degrees of block at shorter CLs. (Data is fit with monoexponential curves τ = 25.42, 8,45 and 2.66 ms for CLs of 330 ms, 1 second and 2 seconds respectively). D. Percent of flecainide-mediated reduction in sodium current amplitude measured after 40 seconds of stimulation at CLs of 330 ms, 1 and 2 seconds for the cells shown in Panel C. E. Representative current recording generated using the protocol shown at baseline and following superfusion with of 1 μM dofetilide with the difference shown in F. This demonstrates the presence of dofetilide-sensitive outward tail current, consistent with the presence of HERG (i.e. I_Kr_) channels in our atrial CMs. (CL=cycle length)

Supplemental Figure 7: Conduction velocity (CV) measurements in atrial-like cells during rotor activity. A. Typical activation map of a CM sheet after rotor induction. B&C. Illustration of CV estimations near the inner core (<5 mm) shown in black versus in regions remote (>50 mm) from the core shown in red. The arrows represent the local direction and magnitude of the electrical activation in the two regions. D. Summary plots of the change in CV recorded before and after the addition of 10μM flecainide in 7 cell sheets. The CV estimates were calculated by averaging all the vector magnitudes in the remote regions (>50 mm from the inner rotor core E. Summary data for CVs estimated by averaging the magnitudes of the CV vectors (shown in Panel C) near the core (black) and at sites remote from the core (blue) before and after the addition of flecainide (5 or 10 μM). * = p<0.05.


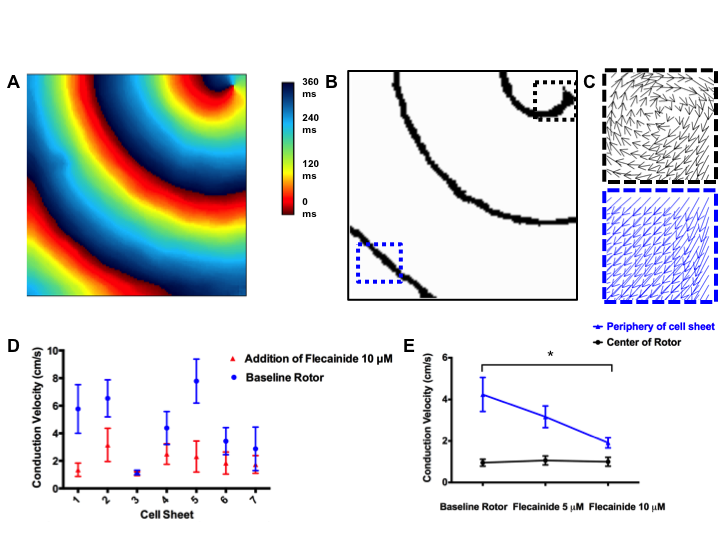


Supplemental Figure 8: Optically mapped action potentials of electrical alternans in the cell sheet during induction of AF through rapid pacing revealed cyclic, beat-to-beat variations in AP amplitude and AP duration when sheets were rapidly stimulated. Immediately following this recording, rotor activity was recorded in the atrial-like cell sheet.


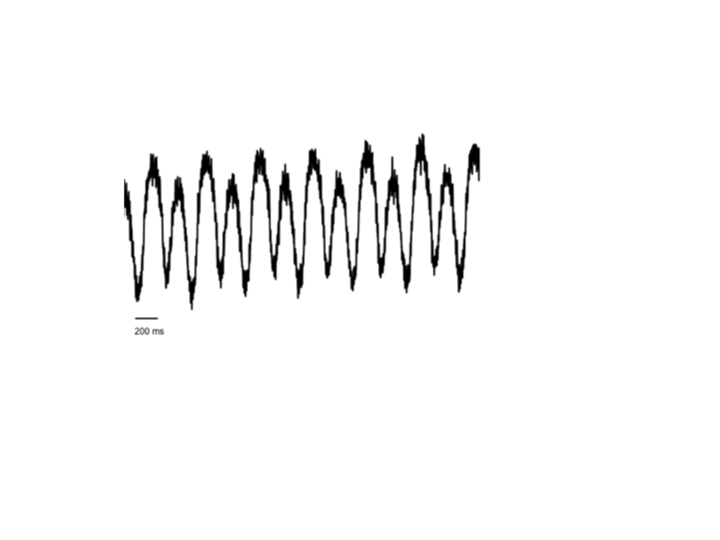


Supplemental Figure 9: An example of an activation maps in cell sheets after rotor induction, demonstrating the ability of flecainide to increase rotor dimensions. On the left, activation map of a rotor induced in an atrial-like cell sheet following rapid pacing protocol. Notice that for this sheet, the single rotor contained 4 activation arms in association with a four phase singularities. On the right, the same cell sheet after the addition of 10 μM flecainide, where now the number of arms associated with the single rotor has been reduced to three.


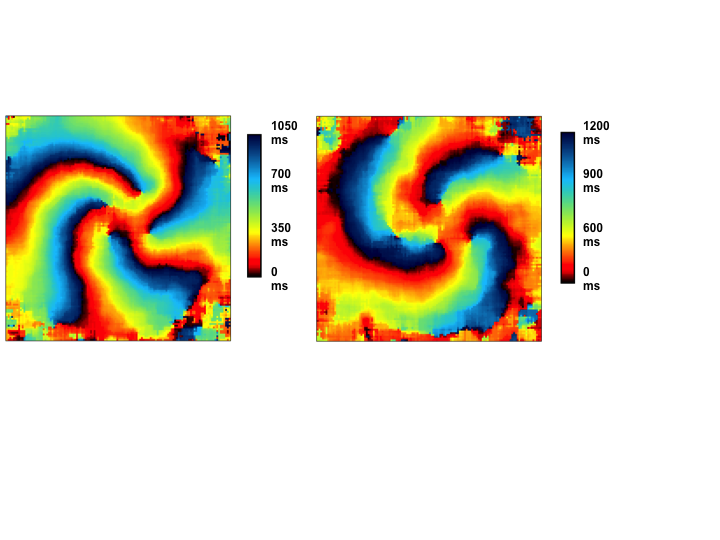


References

1. Kennedy M, D'Souza SL, Lynch-Kattman M, Schwantz S, Keller G. Development of the hemangioblast defines the onset of hematopoiesis in human ES cell differentiation cultures. Blood 2007;**109**(7):2679-87.

2. Thomson JA, Itskovitz-Eldor J, Shapiro SS, Waknitz MA, Swiergiel JJ, Marshall VS, Jones JM. Embryonic stem cell lines derived from human blastocysts. Science 1998;**282**(5391):1145-7.

3. Ng ES, Davis R, Stanley EG, Elefanty AG. A protocol describing the use of a recombinant protein-based, animal product-free medium (APEL) for human embryonic stem cell differentiation as spin embryoid bodies. Nat Protoc 2008;**3**(5):768-76.

4. Elliott DA, Braam SR, Koutsis K, Ng ES, Jenny R, Lagerqvist EL, Biben C, Hatzistavrou T, Hirst CE, Yu QC, Skelton RJ, Ward-van Oostwaard D, Lim SM, Khammy O, Li X, Hawes SM, Davis RP, Goulburn AL, Passier R, Prall OW, Haynes JM, Pouton CW, Kaye DM, Mummery CL, Elefanty AG, Stanley EG. NKX2-5(eGFP/w) hESCs for isolation of human cardiac progenitors and cardiomyocytes. Nat Methods 2011;**8**(12):1037-40.

5. Zhang Q, Jiang J, Han P, Yuan Q, Zhang J, Zhang X, Xu Y, Cao H, Meng Q, Chen L, Tian T, Wang X, Li P, Hescheler J, Ji G, Ma Y. Direct differentiation of atrial and ventricular myocytes from human embryonic stem cells by alternating retinoid signals. Cell Res 2011;**21**(4):579-87.

6. Devalla HD, Schwach V, Ford JW, Milnes JT, El-Haou S, Jackson C, Gkatzis K, Elliott DA, Chuva de Sousa Lopes SM, Mummery CL, Verkerk AO, Passier R. Atrial-like cardiomyocytes from human pluripotent stem cells are a robust preclinical model for assessing atrial-selective pharmacology. EMBO Mol Med 2015;**7**(4):394-410.

7. Dubois NC, Craft AM, Sharma P, Elliott DA, Stanley EG, Elefanty AG, Gramolini A, Keller G. SIRPA is a specific cell-surface marker for isolating cardiomyocytes derived from human pluripotent stem cells. Nat Biotechnol 2011;**29**(11):1011-8.

8. Lin E, Craig C, Lamothe M, Sarunic MV, Beg MF, Tibbits GF. Construction and use of a zebrafish heart voltage and calcium optical mapping system, with integrated electrocardiogram and programmable electrical stimulation. Am J Physiol Regul Integr Comp Physiol 2015;**308**(9):R755-68.

9. Doss MX, Di Diego JM, Goodrow RJ, Wu Y, Cordeiro JM, Nesterenko VV, Barajas-Martinez H, Hu D, Urrutia J, Desai M, Treat JA, Sachinidis A, Antzelevitch C. Maximum diastolic potential of human induced pluripotent stem cell-derived cardiomyocytes depends critically on I(Kr). PLoS One 2012;**7**(7):e40288.
